# Validation of the Finnish version of the Brief International Cognitive Assessment for Multiple Sclerosis (BICAMS) and evaluation of the applicability of the Multiple Sclerosis Neuropsychological Questionnaire (MSNQ) and the Fatigue Scale for Motor and Cognitive Functions (FSMC)

**DOI:** 10.1002/brb3.2087

**Published:** 2021-05-07

**Authors:** Päivi Hämäläinen, Vera Leo, Sebastian Therman, Juhani Ruutiainen

**Affiliations:** ^1^ Masku Neurological Rehabilitation Centre The Finnish Neuro Society Masku Finland; ^2^ Cognitive Brain Research Unit Department of Psychology and Logopedics Faculty of Medicine Helsinki Finland; ^3^ Mental Health Unit Finnish Institute for Health and Welfare Helsinki Finland; ^4^ Department of Neurology University of Turku Turku Finland

**Keywords:** BICAMS, cognition, FSMC, MSNQ, multiple sclerosis, screening

## Abstract

**Objectives:**

Cognitive impairment is frequent in multiple sclerosis (MS) as approximately half of the patients manifest some degree of cognitive impairment. The Brief International Cognitive Assessment for Multiple Sclerosis (BICAMS) has been designed for brief cognitive evaluation. The purpose of the study was to validate the BICAMS along with the Finnish versions of one self‐rating questionnaire each for cognition and fatigue.

**Methods:**

A total of 65 MS patients and 45 healthy controls (HC) were assessed with the BICAMS, the Multiple Sclerosis Neuropsychological Questionnaire (MSNQ), and the Fatigue Scale for Motor and Cognitive Functions (FSMC) twice, approximately within nine days.

**Results:**

MS patients scored markedly lower than the HCs on each of the three tests of the BICAMS. Of the patients, 60% scored at least 1.5 *SD* below the mean of the HCs on at least one test; 49% on the SDMT, 26% on the CVLT‐II, and 28% on the BVMT‐R. Correlation coefficients for the repeated measurement were between 0.75 and 0.89 for the three tests in the whole study sample. MS patients reported more cognitive symptoms and more fatigue than the HCs. Cronbach's alpha was 0.94 for the MSNQ and 0.98 for the FSMC. Correlation coefficient for the repeated measurement was 0.91 for the MSNQ and between 0.92 and 0.94 for the FSMC scores for the whole study sample.

**Conclusions:**

The present study supports the validity of the Finnish version of the BICAMS. The SDMT was the most sensitive of the three BICAMS tests and showed cognitive impairment in half of the patients. The Finnish versions of the MSNQ and the FSMC proved useful tools in approaching concerns related to cognition and fatigue.

## Graphical abstract

A total of 65 MS patients and 45 healthy controls (HC) were assessed with the BICAMS, the Multiple Sclerosis Neuropsychological Questionnaire (MSNQ), and the Fatigue Scale for Motor and Cognitive Functions (FSMC) twice, approximately within nine days. The BICAMS validation was performed according to published recommendations. The results supported the validity of the Finnish version of the BICAMS. The Finnish versions of the MSNQ and the FSMC proved useful tools in approaching concerns related to cognition and fatigue.

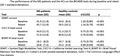

## 1 INTRODUCTION

Cognitive deficits are a common manifestation in multiple sclerosis (MS) occurring in about 50%–60% of patients (Sumowski et al., (2018)). Slowed information processing as well as memory and learning dysfunction are regarded as the most frequent cognitive deficits (Benedict et al., 2020; Sumowski et al., 2018). Slowed information processing speed in particular is thought to be the core feature of cognitive decline in MS. The functional consequences of MS‐related cognitive impairment can be striking (Hämäläinen & Rosti‐Otajärvi, 2014). Cognitive deficits may have effects on physical independence, quality of life, employment, social and recreational activities, driving skills, and rehabilitation outcome, as well as on caregiver strain (Benedict et al., 2020). Since cognitive deficits can have a multidimensional impact on patients' activities of daily living, these symptoms should be considered in the diagnostics and treatment.

Despite the high frequency and obvious negative impact on functioning, cognitive impairment often remains undiagnosed; invisible symptoms, especially mild cognitive impairments, are not observed during routine neurological examinations. To improve the detection of cognitive impairments and to make follow‐up easier, brief assessment tools have been suggested for routine use. An international expert committee agreed on a short battery, the Brief International Cognitive Assessment for Multiple Sclerosis (BICAMS), which is considered a valid and reliable measure of cognitive functioning in MS when comprehensive neuropsychological assessment is not available (Langdon et al., 2012). The BICAMS includes the Symbol Digit Modalities Test (SDMT) (Smith, 1982) evaluating information processing speed, the California Verbal Learning Test (CVLT) (Delis et al., 2000) evaluating verbal memory and learning, and the Brief Visual Memory Test‐Revised (BVMT‐R) (Benedict et al., 1997) evaluating visual memory and learning.

In clinical practice, self‐reports provide an important source of information on subjective symptoms. The Multiple Sclerosis Neuropsychological Questionnaire (MSNQ) (Benedict et al., 2013) has been used to assess cognition‐related concerns. Furthermore, the Fatigue Scale for Cognitive and Motor Functions (FSMC) (Penner et al., 2009) offers a possibility to not only evaluate subjective overall fatigue but also the cognitive and motor components of the symptom. Self‐reports are valuable especially in cases where objective assessment is not available, and they serve as a way to approach a delicate topic. However, self‐reports are vulnerable to different sources of errors and require validation before use in new populations and as new translations. Whereas self‐perceived cognitive and fatigue symptoms have been found to be associated with depression scores, controlling for mood state is necessary.

The primary objective of the present study was to evaluate whether the Finnish BICAMS is a valid measure of cognitive status in MS by employing the validation procedure suggested by Benedict et al. (2012). The secondary aim was to evaluate the applicability of the Finnish translation of the MSNQ and the FSMC in patients with MS.

## 2 MATERIALS AND METHODS

### 2.1 Participants

The participants of the present study consisted of 65 patients with MS and 45 healthy controls (HC). The patients were recruited during 2018 to 2019 from Masku Neurological Rehabilitation Centre in Finland. The study was approved by the Ethical Committee of the Hospital District of Southwest Finland and was performed in conformance with the Declaration of Helsinki (World Medical Association, 2013). All participants gave informed, written consent. All patients were diagnosed with MS according to the 2010 revised McDonald criteria (Polman et al., 2011). The other inclusion criteria were age between 18 and 65 years, Finnish as a native language, adequate visual acuity, and audition based on interview, no reported alcohol or substance abuse, no other neurological illness except MS, no severe psychiatric illness, no primary learning disability, and no relapse during one month prior to the study. The medical records of patients with MS were prescreened for inclusion criteria. After this prescreening, 73 patients with MS were informed of the study and eight of them refused to participate, mainly due to unwillingness to be assessed. HCs were recruited from the personnel of the rehabilitation center as well as their relatives and friends following the inclusion and exclusion criteria described except for those related to multiple sclerosis. A total of 65 HCs were given the study information; five of them did not fulfill the inclusion criteria, 14 refused to participate mainly due to unwillingness to be assessed, and one was not willing to continue after the baseline assessment. Age, gender, educational degree, years of education, employment status, and mood state were recorded for all the participants. Disease duration, disease subtype, and the Patient‐Reported Expanded Disability Scale (PREDSS; 13) were recorded for the patients with MS.

### 2.2 BICAMS test battery

The study procedures followed the recommendations for the BICAMS national validation including (a) standardization and translation of test stimuli, (b) standardization and translation of test instruction, (c) normalization, (d) evaluation of test–retest reliability, and (e) evaluation of criterion‐related validity (Benedict et al., 2012). All the participants performed the BICAMS tests and completed the questionnaires on cognition, fatigue, and mood twice, at baseline and after approximately nine days later.

### 2.3 SDMT

The Symbol Digit Modalities Test (SDMT; 5) measures the speed of information processing. The test consists of a sheet with nine symbols presented in pseudo‐randomized lines. Each symbol is paired with a digit 1–9 in a key at the top of the sheet. The participant is asked to pair, in order, as many of the symbols to the corresponding digits as they can in 90 s. The existing Finnish version of the test and the instructions was employed, and same version of the test was used in both assessments. The number of orally given correct answers during 90 s served as the dependent variable.

### 2.4 CVLT‐II

The California Verbal Learning Test II (CVLT‐II; 6) measures verbal learning. The immediate recall consists of five learning trials of a word list of four words each in four semantic categories. The examiner reads the words aloud at a steady pace during 20 s. The participant listens to the complete list and is asked to recall as many words as possible in any order. The list of 16 words had previously been adapted and standardized into Finnish (Vuorivirta, 2006). The same test version was used in both assessments. An alternate version of the CVLT‐II is not available in Finnish. The dependent variable was the total number of words recalled during the five trials.

### 2.5 BVMT‐R

The Revised version of the Brief Visuospatial Memory Test (BVMT‐R; 7) measures visual learning. The test consists of six abstract symbols on a sheet of paper. Participants are given 10 s to look at the symbols and are then asked to draw as many symbols as they can recall in the right order on an empty sheet of paper. Performance is scored on accuracy and location with 0–2 points per symbol. The task is repeated three times. For the present study, the existing Finnish version of the test and the instructions were employed. There are several alternative forms of the test; version 1 was used during baseline and version 2 during retest. The sum score on the three trials served as the dependent variable.

### 2.6 2.3. Self‐rating questionnaires

Subjective cognitive complaints were assessed by using the Finnish version of the MSNQ (Benedict et al., 2013), which consists of 15 questions assessing cognitive restrictions with the scale ranging from 0 (never) to 4 (frequently). The total score served as the dependent variable. Subjective feelings of fatigue were evaluated with the FSMC (Delis et al., 2000). The questionnaire consists of 20 statements related to motor and cognitive aspects of fatigue with the scale ranging from 1 (totally disagree) to 5 (totally agree). The total score as well as the sub‐scores for motor and cognitive fatigue served as the study variables. Mood was assessed CES‐D questionnaire (Radloff, 1977).

### 2.7 Statistical analyses

Groups were compared with the Mann–Whitney *U* test and the Wilcoxon test for continuous and ordered variables and the chi‐square test for binary variables. Results were considered statistically significant when *p* <.05, without correction for multiple testing. Group differences were quantified using the Common Language Effect Size statistic (CLES; McGraw & Wong, 1992) and Cohen's d (Cohen, 1988). Relationships between the study variables and test–retest reliability were evaluated with Spearman rank order correlations. The test–retest reliability was considered acceptable when the correlation coefficient was greater than 0.70. Performance on individual tests was considered impaired if at or below the −1.5 *SD* level of the HC distribution (Sumowski et al., 2018). Overall cognitive performance was defined as impaired if performance at least on one test of the BICAMS was impaired. The internal consistency of the MSNQ and the FSMC questionnaires was evaluated with Cronbach's alpha, with 0.70 considered acceptable. Statistical analyses were performed with IBM SPSS 24.0.

### 2.8 Results

The background variables of the study groups are reported in Table 1. The mean interval of the baseline and the retest was 9.0 (*SD* 3.4) days. The study groups were statistically similar with respect to gender, age, and years of education, but differed in employment status and self‐rated mood state. Based on the PREDSS, 28% of the patients had mild to moderate disability (EDSS 0–3), 61% severe disability (4–6.5), and 11% were restricted to a wheelchair (Benedict et al., 1997; Benedict et al., 2013; Penner et al., 2009). A majority of the patients (62%) had a relapsing–remitting and a minority (38%) a progressive form of the disease. There were no missing values in the data.

**TABLE 1:** Baseline characteristics of the MS patients and the HCs (*SD* = standard deviation)

	MS patients (*n* = 65)	Healthy controls (*n* = 45)	*p*
Female; % (*n*)	71.0 (46)	71.0 (32)	0.97
Age, years; mean (*SD*)	50.9 (8.8)	49.4 (12.6)	0.35
Education, years; mean (*SD*)	13.8 (9.8)	14.0 (2.1)	0.69
Employment status			<0.001
Employed; % (*n*)	20.0 (13)	86.7 (39)	
Disability pension; % (*n*)	67.7 (44)	0.0 (0)	
Other condition; % (*n*)	12.3 (8)	13.3 (6)	
Disease duration, years since symptoms; mean (*SD*)	21.9 (11.2)	—	
Disease duration, years since diagnosis; mean (*SD*)	15.9 (9.8)	—	
PREDSS score (range 0–9); mean (*SD*)	4.8 (2.0)	—	
Mood state, CES‐D total (range 0–60); mean (*SD*)	11.5 (7.6)	6.6 (5.8)	0.001

Abbreviations: CES‐D, Center for Epidemiologic Studies Depression Scale; PREDSS, Patient‐Reported Expanded Disability Status Scale.

The MS patients scored significantly lower than the HCs on each single test of the BICAMS both at the baseline as well as at the retest (Table 2). The between‐groups Cohen's ds were from 0.69 to 1.20 showing medium to very large effect sizes (Cohen, 1988). Both groups showed practice effects as the performance at the retest exceeded that observed at the baseline.

**TABLE 2:** The performance of the MS patients and the HCs on the BICAMS tests during baseline and retest (*SD* = standard deviation)

Test	MS patients mean (*SD*)	Healthy controls mean (*SD*)	*d* (CLES)	*p*
SDMT correct				
Baseline	41.9 (11.8)	54.6 (8.3)	1.20 (0.80)	<0.001
Retest	45.7 (12.9)	59.5 (10.1)	1.16 (0.79)	<0.001
CVLT‐II total score				
Baseline	43.0 (11.5)	51.3 (10.7)	0.75 (0.70)	<0.001
Retest	51.6 (13.8)	60.8 (12.0)	0.70 (0.69)	<0.001
BVMT‐R total score				
Baseline	19.2 (8.0)	24.7 (6.8)	0.73 (0.70)	<0.001
Retest	20.8 (7.2)	25.3 (5.4)	0.69 (0.69)	<0.001

Abbreviations: BVMT‐R, Brief Visual Memory Test‐Revised; CLES, The Common Language Effect Size statistic; CVLT‐II, California Verbal Learning Test‐II; d, Cohen's *d* with pooled standard deviation; SDMT, Symbol Digit Modalities Test.

At baseline, 60% (39/65) of the patients were impaired on at least one of the three BICAMS tests. Of the patients, 29% (19/65) showed impaired performance on one test, 19% (12/65) on two tests, and 12% (8/65) on all three tests. The SDMT was the most sensitive test of the BICAMS as almost half of the patients had impaired performance (Table 3).

**TABLE 3:** MS patients performing at least 1.5 standard deviations (*SD*) below the mean of the HCs at baseline

Test	Cut‐off (score equal to or more than 1.5 *SD* below the mean of the HCs)	MS patients impaired, performance at or under the cut‐off % (*n*)	MS patients impaired also on another test of the BICAMS % (*n*)
SDMT correct	42	49 (32)	59 (19)
CVLT‐II total score	35	26 (17)	76 (13)
BVMT‐R total score	14	28 (18)	89 (16)

Abbreviations: BVMT‐R, Brief Visual Memory Test‐Revised; CVLT‐II, California Verbal Learning Test‐II; SDMT, Symbol Digit Modalities Test.

MS patients reported more subjective cognitive complaints as well as feelings of motor and cognitive fatigue than the HCs both at the baseline and at the retest as seen as significantly higher scores on the MSNQ and the FSMC (Table 4). The between‐groups Cohen's *d*s were over 1.0 showing at least large effect sizes (Cohen, 1988). Cronbach's alpha for the MSNQ was 0.94, for the whole FSMC 0.98, and for both the cognitive and the motor subscales of the FSMC 0.96.

**TABLE 4:** The results of MS patients and HCs on the MSNQ and the FSMC during baseline and retest (*SD* = standard deviation)

Test	MS patients mean (*SD*)	Healthy controls mean (*SD*)	*d* (CLES)	*p*
MSNQ‐T				
Baseline	23.9 (11.1)	13.8 (6.6)	1.06 (0.77)	<0.001
Retest	25.4 (11.1)	12.2 (6.3)	1.39 (0.84)	<0.001
FSMC‐T				
Baseline	49.2 (16.7)	9.3 (8.4)	2.87 (0.98)	<0.001
Retest	48.2 (16.7)	8.8 (8.4)	2.82 (0.98)	<0.001
FSMC‐M				
Baseline	25.7 (8.2)	3.9 (4.4)	3.16 (0.99)	<0.001
Retest	25.3 (8.0)	3.6 (3.5)	3.29 (0.99)	<0.001
FSMC‐C				
Baseline	23.5 (9.9)	5.4 (4.5)	2.22 (0.94)	<0.001
Retest	22.8 (9.8)	5.2 (5.5)	2.11 (0.93)	<0.001

Abbreviations: CLES, The Common Language Effect Size statistic; d, Cohen's *d* with pooled standard deviation; FSMC‐C, FSMC cognitive sub‐score; FSMC‐M, FSMC motor sub‐score; FSMC‐T, Fatigue Scale for Motor and Cognitive Functions, total score; MSNQ‐T, Multiple Sclerosis Neuropsychological Questionnaire, total score.

Correlations between the study variables, the BICAMS, the MSNQ, the FSMC, and the CES‐D are presented in Table 5. Of the MS patients, 46% (30/65) reported subjective cognitive complaints as manifested as a total score equal to or over 27 points (Benedict et al., 2013); 63% (19/30) of those patients showed impairment on at least one of the tests of the BICAMS and 57% (17/30) specifically on the SDMT. Of the patients who reported cognitive complaints, 30% did not show impairment on any of the BICAMS tests. The correlation between the MSNQ and the CES‐D was found to be moderately positive and significant (Table 5). Some 69% (45/65) of the MS patients reported at least mild overall fatigue as manifested as a total score equal to or over 43 points (Penner et al., 2009), while 77% (50/65) of the MS patients reported at least mild motor fatigue (motor sub‐score ≥ 22 points) and 62% (40/65) at least mild cognitive fatigue (cognitive sub‐score ≥ 22). Of patients who reported at least mild cognitive fatigue on the FSMC (≥22 points), 58% (23/40) showed impairment on at least one of the tests of the BICAMS battery and 50% (20/40) specifically on the SDMT. Correlation between the FSMC scores and the CES‐D total score is presented in Table 5.

**TABLE 5:** Correlations between the BICAMS test scores, the MSNQ total score, the FSMC total score and sub‐scores, and the CES‐D total score in the MS group at baseline

	SDMT	CVLT‐II	BVMT‐R	MSNQ	FSMC‐T	FSMC‐M	FSMC‐C
CVLT‐II	0.46***						
BVMT‐R	0.61***	0.58***					
MSNQ‐T	−0.26**	−0.12	−0.11				
FSMC ‐T	−0.51***	−0.27**	−0.29**	0.76***			
FSMC‐M	−0.55***	−0.25**	−0.31**	0.67***	0.96***		
FSMC‐C	−0.45***	−0.29**	−0.27**	0.78***	0.97***	0.87**	
CES‐D	−0.26**	0.09	−0.16	0.48**	0.48***	0.48**	0.45***

Abbreviations: CES‐D, Center for Epidemiologic Studies Depression Scale; FSMC‐C, FSMC cognitive sub‐score; FSMC‐M, FSMC motor sub‐score; FSMC‐T, Fatigue Scale for Motor and Cognitive Functions, total score.

* *p* < .05

** *p* < .01

*** *p* < .001

Test–retest reliability results are reported in Table 6. All the test–retest correlations for the whole study sample as well as for the MS patients were over 0.70 (all *p* < .001). In the HC group, the test–retest correlations for the FSMC motor sub‐score remained under 0.70.

**TABLE 6:** Correlations between baseline and retest for the BICAMS tests, the MSNQ, and the FSMC

Test	Whole sample (*n* = 110)	MS patients (*n* = 65)	Healthy controls (*n* = 45)
	*ρ*	*ρ*	*ρ*
SDMT	0.89	0.86	0.86
CVLT‐II	0.83	0.84	0.78
BVMT‐R	0.75	0.71	0.75
MSNQ	0.91	0.89	0.84
FSMC‐T	0.94	0.87	0.72
FSMC‐M	0.92	0.79	0.64
FSMC‐C	0.92	0.86	0.73

Abbreviations: BVMT‐R, Brief Visual Memory Test‐Revised; CVLT‐II, California Verbal Learning Test‐II; FSMC‐C, FSMC cognitive sub‐score; FSMC‐M, FSMC motor sub‐score; FSMC‐T, Fatigue Scale for Motor and Cognitive Functions, total score; MSNQ‐T, Multiple Sclerosis Neuropsychological Questionnaire, total score; SDMT, Symbol Digit Modalities Test.

## 3 DISCUSSION

The aim of the present study was to validate the BICAMS in a Finnish population with MS by employing the validation procedure suggested by Benedict et al. (2012). The secondary aim of the study was to evaluate the applicability of the Finnish version of the MSNQ (Benedict et al., 2013) and the FSMC (Penner et al., 2009) in Finnish patients with MS.

MS patients performed significantly worse than the HCs on each three tests of the BICAMS. On the SDMT, the difference between the HC and the MS group at baseline was almost 13 points whereas the difference has varied from nine to 16 in other studies (Costers et al., 2017; Dusankova et al., 2012; Filser et al., 2018; Giedraitiene et al., 2015; Niino et al., 2017; O’Connell et al., 2015; Ozakbas et al., 2017; Polychroniadou et al., 2016; Sandi et al., 2015; Spedo et al., 2015; Vanotti et al., 2016). Similarly, the difference between the two groups was more than eight points on the CVLT‐II at the baseline in the present study, while it has varied between one to 10 points in other studies (Costers et al., 2017; Dusankova et al., 2012; Giedraitiene et al., 2015; Niino et al., 2017; O’Connell et al., 2015; Ozakbas et al., 2017; Polychroniadou et al., 2016; Sandi et al., 2015; Spedo et al., 2015; Vanotti et al., 2016; Walker et al., 2016). On the BVMT‐R, the difference between the HCs and the MS patients was over five points at baseline while it has been between three and six points in other studies (Costers et al., 2017; Dusankova et al., 2012; Filser et al., 2018; Giedraitiene et al., 2015; Niino et al., 2017; O’Connell et al., 2015; Ozakbas et al., 2017; Polychroniadou et al., 2016; Sandi et al., 2015; Spedo et al., 2015; Vanotti et al., 2016; Walker et al., 2016). Our participants were older than those in the other studies. Furthermore, they had longer disease duration, more severe disability, and more often a progressive form of MS compared with the other studies. These features probably explain the relatively big differences in the test scores between the MS patients and the HCs as well as the lower overall performance compared with most of the other BICAMS studies.

Both groups showed practice effects on the tests of the BICAMS. The performances at the retest exceeded those observed at the baseline in both groups. The differences in practice effects between the groups were small. The same versions of the SDMT and the CVLT‐II tests were used for the repeated measurements. Instead, parallel versions were used for the BVMT‐R. The practice effects can be suggested to be more evident when same test version is repeated than when parallel versions are applied. This was also the case in the present study the difference on the SDMT being 3.9 points for the MS group and 4.9 for the HCs, on the CVLT‐II 8.7 and 9.5 points, and on the BVMT‐R 1.6 and 0.6 points, respectively. In an Italian study by Goretti et al. (Goretti et al., 2014) with a sample of 243 HCs tested twice, the baseline performance was slightly better than in the HCs of the present study. The finding is probably due to the fact that their patients were 11 years younger and slightly more educated compared with ours. The difference between the baseline and the retest in the Italian study was 4.1 points for the SDMT, 8.0 points for the CVLT‐II, and 3.1 points for the BVMT‐R. The differences were relatively similar to those observed in the present study despite the bigger difference in the BVMT‐R which is probably explained by the use of same test version twice in the Italian study. Furthermore, linguistic and cultural differences that occur in test translations as well as differences in the time the tests are repeated may explain the subtle differences in the test results between different language versions.

Altogether 60% of the patients showed impaired performance on at least one of the BICAMS tests. This finding is well in line with the known frequency of cognitive impairment in MS (Benedict et al., 2020; Sumowski et al., 2018) as well as with previous findings with the BICAMS, for example, Canadian and Czech population showed the impairment rate of 58% (Dusankova et al., 2012; Walker et al., 2016), Irish 57% (O’Connell et al., 2015), and Hungarian 52% (Sandi et al., 2015). The Finnish version of the BICAMS seems to tap MS‐related cognitive impairment at a satisfactory level and, thus, can be considered as a useful and valid measure to identify MS patients who may have cognitive impairments.

From the three single tests of the Finnish version of the BICAMS, the SDMT was the most sensitive followed by the BVMT‐R and the CVLT‐II, showing impairment rates of 49%, 28%, and 26%, respectively. O’Connell and colleagues (O’Connell et al., 2015) reported an impairment rate of 37% for the SDMT, 10% for the BVMT‐R, and 40% for the CVLT‐II using the same criteria as used in the present study. Polycroniadou and colleagues (Polychroniadou et al., 2016) reported an impairment rate of 43% for the SDMT, 22% for the BVMT‐R, and 20% for the CVLT‐II using the 5th percentile as a cut‐off score. Our results corroborate the earlier findings on the sensitivity of the SDMT. The SDMT has been suggested as the most sensitive single task to tap MS‐related cognitive deficits, especially those related to processing slowness (Benedict et al., 2017; López‐Góngora et al., 2015).

The test–retest reliability of the BICAMS was evaluated with the correlation coefficients. For the SDMT as well as the CVLT‐II, the correlations for the whole study sample as well as for the MS group were > 0.80 indicating good test–retest reliability. For the BVMT‐R, the correlation was > 0.70 showing adequate test–retest reliability. These results are in line with the findings from the other BICAMS validation studies in which the correlations for the SDMT and the CVLT‐II have been higher than those for the BVMT‐R (Filser et al., 2018; Goretti et al., 2014; Niino et al., 2017; Walker et al., 2016). Our results also show that the translation and adaptation of the California Verbal Learning Test into Finnish is appropriate and has a good test–retest reliability.

Unsurprisingly, MS patients reported significantly more cognitive complaints than the HCs on the MSNQ. Altogether 46% of the patients reported subjective cognitive complaints with a total score equal to or over 27 points (Benedict et al., 2008). To compare, 63% of them showed impairment on at least one of the tests of the BICAMS battery and 57% specifically on the SDMT. A third of the patients who reported cognitive complaints did not show impairment on any of the BICAMS tests. The MSNQ showed high internal consistency. The correlation between the total score of the MSNQ and the SDMT was negative and statistically significant, whereas the correlation between the MSNQ and the CVLT‐II, and the BVMT‐R were statistically non‐significant. Instead, correlations between the total score of the MSNQ and the total score as well as sub‐scores of the FSMC, and the CES‐D were all statistically significant, supporting the earlier findings that low mood state and other symptoms, like fatigue may explain patients’ cognitive complaints (Benedict et al., 2008; O’Brien et al., 2007). The test–retest reliability of the MSNQ was good, the correlations for the whole study sample as well as for the MS group and HCs separately being > 0.80, as observed also previously (Benedict et al., 2008; Morrow et al., 2010). The results of the present study confirm the earlier findings that the MSNQ score is related to the elevated scores in depression questionnaires (Benedict et al., 2008; O’Brien et al., 2007) and, thus, should be used together with an evaluation of mood state. The MSNQ might better serve as a tool to approach this delicate topic than as a screening instrument for cognition per se.

MS patients reported significantly more fatigue than HCs on the FSMC. Altogether, 69% of the patients reported at least mild overall fatigue (Penner et al., 2009) with a total FSMC score equal to or over 43 points. Mild or worse motor fatigue (motor sub‐score ≥ 22) was reported by 77% and mild or worse cognitive fatigue (cognitive sub‐score ≥ 22) by 62% of patients. These findings are in line with the known prevalence of MS fatigue, which is up to 83% (Manjaly et al., 2019). The FSMC showed high internal consistency as Cronbach's alpha was over 0.95 for the total as well as for the sub‐scales. Altogether 58% of our patients who reported at least mild cognitive fatigue on the FSMC (≥22 points) showed impairment on at least one of the tests of the BICAMS battery and 50% specifically on the SDMT. The correlations between the total and the sub‐scores of the FSMC and the SDMT, the CVLT‐II and the BVMT‐R were all negative and statistically significant. In contrast, the correlation between the cognitive sub‐score of the FSMC and the MSNQ was positive and statistically significant. The test–retest correlations of the FSMC scores of > 0.90 for the whole sample were excellent. For the MS group, the FSMC total score and the cognitive sub‐score showed good test–retest reliability. Only the test–retest correlation of the FSMC motor sub‐score in the HC group remained under 0.70. The Finnish version of the FSMC seems to serve as a potentially useful method for identification and follow‐up of MS patients’ fatigue symptoms.

The present study followed the recommended BICAMS validation procedure in a sample of 65 MS patients and 45 HCs, tested twice within a short interval in controlled study environment. The median duration of MS was 15 years from diagnosis and the patient reported disability score (PREDSS) (Kobelt et al., 2006) was approximately 5.0. These features explain the slightly elevated cognitive impairment rate in our study compared with other studies using the BICAMS for younger patients with milder disability. We used the existing Finnish versions of the SDMT and the BVMT‐R which both showed appropriate test–retest validity. The CVLT‐II was translated and standardized using recommended procedures which resulted in a test version (Vuorivirta, 2006) with good test–retest reliability. In the validation, we used the same versions of the SDMT and the CVLT‐II during baseline and retest as suggested in the original validation procedures (Benedict et al., 2012). To evaluate how similar the parallel forms of the BVMT‐R are, two different versions were used. The use of parallel versions probably explains a smaller practice effect than observed in the other BICAMS studies using a single test version. For follow‐up purposes, parallel versions might be preferred if test–retest validity has been established. In the present study, we could have used the alternate version of the SDMT but not that of the CVLT‐II because such does not exist in Finnish. We also evaluated the applicability of the existing Finnish versions of the MSNQ and the FSMC. Both self‐rating questionnaires were easy to administer, showed good internal consistency and adequate test–retest reliability, especially in the MS group. Self‐perceived cognitive problems as evaluated by the MSNQ and self‐perceived fatigue as evaluated by the FSMC were associated with lowered mood state. Therefore, both questionnaires should be used together with the evaluation of mood state. More detailed validation of the MSNQ and the FSMC will require a larger sample size and an additional fatigue scale to evaluate the criterion validity. Thus, the results on the two questionnaires have to be considered preliminary.

## CONFLICT OF INTEREST

None of the authors have any conflict of interest related to this study. P Hämäläinen belonged to the International Committee which originally suggested the BICAMS to be used in cognitive evaluation of patients with MS.

## AUTHOR CONTRIBUTION

Hämäläinen involved in planning the study, acquiring the funding, administration of the project, permission for the validation from the test publishers and authors, supervision, analyzing the data, and writing and editing the manuscript. Leo involved in data collection, data curation, statistical analysis, formatting the tables, and editing the manuscript. Therman involved in statistical analysis, formatting the tables, and editing the manuscript. Ruutiainen involved in planning the study, neurological expertise, and editing the manuscript.

### PEER REVIEW

The peer review history for this article is available at https://publons.com/publon/10.1002/brb3.2087.

## Data Availability

The data collected in the present study can be shared only if permitted by the local ethics committee.
